# Psychosocial impact of alopecia areata in paediatric and adolescent populations: A systematic review

**DOI:** 10.1111/jpc.16678

**Published:** 2024-10-07

**Authors:** Isabella J Tan, Mohammad Jafferany

**Affiliations:** ^1^ Department of Dermatology Rutgers Robert Wood Johnson Medical School New Brunswick New Jersey USA; ^2^ Department of Psychiatry and Behavioral Sciences Central Michigan University College of Medicine Mount Pleasant Michigan USA

**Keywords:** adolescent, alopecia areata, paediatric, psychodermatology, psychological

## Abstract

Alopecia areata is an autoimmune disorder characterised by sudden hair loss, and can range from patchy baldness to more severe forms such as alopecia totalis and universalis. Hair loss can have a profound impact on self‐esteem and body image, particularly during childhood and adolescence. Understanding the psychosocial impact of alopecia areata in paediatric and adolescent populations is crucial to address the emotional and social challenges faced by these patients. The aim is to review the existing literature for clinical studies and reports investigating the psychosocial impact of alopecia areata in paediatric and adolescent populations. A systematic review of the literature was performed using PubMed, Cochrane and Embase databases from inception to July 2023. Included articles assessed the psychosocial impact of alopecia areata in paediatric and adolescent populations. Of 79 total articles, 10 were identified as meeting the inclusion criteria. Several studies highlighted self‐esteem, emotional distress and social challenges as features of psychosocial manifestations. Factors such as stress, psychiatric comorbidities and familial issues are significantly associated with alopecia areata in these populations. The heterogeneity of studies precluded data synthesis and analysis. A majority of the included studies evaluated short‐term findings. Alopecia areata has significant psychosocial impacts in paediatric and adolescent populations, with studies emphasising the negative effects on self‐esteem, body image and quality of life. Additional research is required to better elucidate this relationship and draw meaningful conclusions to guide clinical support and interventions.

**Key points**

1. Paediatric and adolescent populations affected by alopecia areata face significant psychosocial challenges, including impaired self‐esteem, heightened stress, anxiety, depression, social stigma and diminished quality of life, necessitating tailored interventions to address their diverse psychological needs.
2. Despite methodological variability among studies, this systematic review identified a consistent pattern of psychosocial impacts of alopecia areata in young populations. Insights emphasise the urgency for individualised care approaches and underscore the need for further research to deepen understanding and develop effective interventions.
3. Holistic approaches encompassing both physical and emotional well‐being are essential to mitigate the psychosocial impact of alopecia areata. Collaboration among health‐care professionals, psychologists, educators and support organisations is paramount to develop comprehensive support systems and improve outcomes for affected individuals.

Alopecia areata (AA), an autoimmune disorder, ranges from patchy baldness to more severe forms such as alopecia totalis (total scalp hair loss) and alopecia universalis (total body hair loss, including eyebrows and eyelashes), posing significant concerns among paediatric and adolescent populations.^1^ The onset of this condition frequently occurs during childhood or adolescence, making it particularly relevant for these age groups.^1^ Epidemiological studies estimate the prevalence of AA in paediatric and adolescent populations to be approximately 0.1%, underscoring the importance of understanding its psychosocial impact in these vulnerable populations.^2^

Hair loss can have a profound and lasting impact on the self‐esteem and body image of affected individuals, especially during the developmental stages of childhood and adolescence.^3^ The loss of hair can elicit negative emotional responses, leading to heightened self‐consciousness and reduced confidence in social interactions.^3^ Consequently, the psychosocial implications of AA can manifest as significant emotional distress, social isolation, and impaired quality of life in paediatric and adolescent patients.

Understanding and addressing the psychosocial impact of AA in these populations is essential, as it involves unique emotional and social challenges. The field of psychodermatology plays a crucial role in comprehending and managing these impacts by integrating both dermatological and psychological approaches.^4^, ^5^ Psychodermatologists provide support, counselling and therapy to help patients cope with their individual stressors and manifestations associated with AA.

Treatment for AA should account for the emotional distress, body image concerns and social challenges that may be experienced by patients. Dermatologists should be aware of these factors, especially in these vulnerable populations, and incorporate tools such as the Dermatology Life Quality Index and Child Dermatology Life Quality Index (CDLQI) into their practice. By incorporating these tools, dermatologists can tailor therapies and interventions to address the specific psychological needs of their patients, contributing to more effective and targeted care.

Through this comprehensive review, the psychosocial challenges faced by young individuals with AA will be highlighted, emphasising the importance of recognising and addressing these aspects in clinical practice, research endeavours and the development of supportive interventions. By synthesising and critically evaluating previous studies, the emotional and social consequences of AA will be explored and elucidated in these age groups. Additionally, the significance of addressing the psychosocial aspects of the condition will be considered in order to foster a holistic approach in the clinical management and support provided to these populations.

## Methods

This systematic review followed Preferred Reporting Items for Systematic Reviews and Meta‐Analyses (PRISMA) guidelines. To identify literature from inception to 7 July 2023, the following search strategy was used within the PubMed, Cochrane and Embase databases: (‘pediatric alopecia areata’ OR ‘adolescent alopecia areata’) AND (‘psychosocial’). Studies were included if they directly measured or evaluated some aspect of the psychosocial impact of AA in paediatric and adolescent populations. Studies were excluded if they were not published in English or did not directly evaluate or measure any aspect of the psychosocial impact of AA in paediatric and adolescent populations. An independent reviewer (IJT) screened titles and abstracts, followed by full‐text articles. The following data were extracted from articles meeting inclusion criteria, when available: study design, participant demographics, measurement tools used and findings related to the psychosocial impact of AA in paediatric and adolescent populations.

## Results

**Fig. 1:**
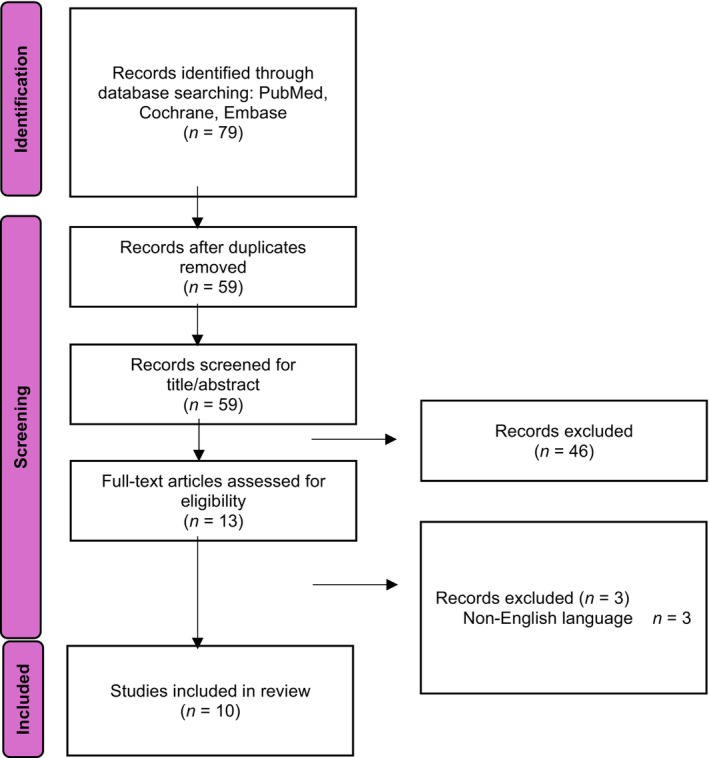
Flow diagram of the systematic study selection process. Source: Page *et al*.^6^ For more information, visit: http://www.prisma‐statement.org/

The PRISMA flow diagram is available in Figure 1. In total, 79 records were identified and, after the removal of duplicates, 59 were screened. Overall, 10 articles met the inclusion criteria and were summarised in this review. Four studies employed a qualitative interview approach, and the remaining six utilised case–control, cross‐sectional and questionnaire study designs. Included studies were published between October 2008 to February 2023. Four studies were from the USA, and 1 each from India, Turkey, Taiwan, Pakistan, Spain and Finland.

Patient characteristics, study design, psychosocial measurement tool and statistically significant psychosocial impacts are outlined in Table 1. The average age of the patients was 12 years (range, 2–19 years).

**Table 1:** Included studies evaluating the psychosocial impact of alopecia areata in paediatric and adolescent populations

Author	Study type	Patient characteristics	Psychosocial measurement tool	Statistically significant psychosocial impact
Benton *et al*.^7^	Questionnaire	32 females, 18 males, ages 7–17 years	Self‐reported questionnaire measuring changes in desired activity participation and psychosocial distress	Impaired self‐esteem Decreased social participation
Saraswat *et al*.^8^	Case–control	49 females, 53 males, ages 2–14 years	Unstructured interview identifying stress arising from personal or familial conditions, school‐related issues and psychotrauma or illness	Stress (school‐related, family‐related)
Bilgiç *et al*.^9^	Cross‐sectional	74 children, ages 8–18 years	Unstructured interview to evaluate psychiatric status and health‐related quality of life (HRQL)	Anxiety‐impaired HRQL
Macey *et al*.^10^	Qualitative interview	11 adolescents, ages 12–17 years	Semi‐structured, combined concept elicitation and cognitive recorded interviews. Transcripts underwent thematic and framework analysis.	Loss of identity Self‐consciousness Social challenges
Chu *et al*.^11^	Case–control	90 patients, ages 2–19 years	Data obtained from the National Health Insurance Research Database of Taiwan from 2000 to 2009. Subgroup analysis for patients between 2 and 19 years.	Depression Anxiety disorders Adjustment disorder
Ghanizadeh^12^	Qualitative interview	14 patients, mean age 11 years	Semi‐structured interview using the *Diagnostic and Statistical Manual of Mental Disorders‐Fourth edition* (DSM‐IV) criteria	Major depressive disorder Anxiety disorders (obsessive‐compulsive disorder)
Rafique and Hunt^13^	Qualitative interview and interpretive analysis	8 adolescents, ages 15–19	Semi‐structured interview, followed by interpretative phenomenological analysis	Loss of self‐identity Social stigma Maladaptive coping style
Díaz‐Atienza and Gurpegui^14^	Qualitative interview	15 females, 16 males, ages 7–19 years	Semi‐structured qualitative interview on stressful life events in the 12 months preceding AA onset and of developmental and family conditions	Perceived stress Decreased expressiveness
Savaş Erdoğan *et al*.^15^	Cross‐sectional	31 patients, ages 7–17 years and their parents	Patients completed the Revised Child Anxiety and Depression Scales‐Child version (RCADS‐C), and their parents completed the parent version (RCADS‐P)	Generalised anxiety Social phobia Major depressive disorder Parental psychological distress (anxiety)
Liu *et al*.^16^	Questionnaire	40 females, 21 males, mean age 10 years, and their parents	Self‐reported questionnaire containing the HRQL instruments Children's Dermatology Life Quality Index (CDLQI) for ages 4–16 and Family Dermatology Life Quality Index (FDLQI) for adult family members	Impaired HRQL (emotional distress, decrease in self‐perception and social functioning) Parental psychological distress (anxiety, depression)

Several studies have investigated the psychosocial impact of paediatric AA, highlighting the challenges and implications for affected individuals. Variability in study design and reported data among articles precluded synthesis and analysis. The questionnaire studies conducted by Benton *et al*. and Liu *et al*. examined the psychological effects of AA through self‐reported measures. Benton *et al*. identified that 75% of participants reported a negative impact on their self‐esteem, and 60% experienced teasing or bullying due to hair loss. Liu *et al*. demonstrated that the severity of AA was associated with greater impairment in health‐related quality of life (HRQL) for both individuals with the condition and their family members. The case–control studies by Saraswat *et al*. and Chu *et al*. investigated the associations between AA and various psychosocial factors. Saraswat *et al*. revealed a significant association between stress related to school issues and familial issues with the onset and exacerbation of AA. Chu *et al*. observed a higher prevalence of psychiatric comorbidities, such as depression, anxiety disorders and adjustment disorder, in patients with AA compared to the control group. The cross‐sectional studies by Bilgiç *et al*. and Savaş Erdoğan *et al*. explored the impact of AA on HRQL and the psychological well‐being of patients and their parents. Bilgiç *et al*. found a correlation between the severity of AA and HRQL impairment, while Savaş Erdoğan *et al*. reported significantly higher anxiety levels in parents of paediatric patients with AA. The qualitative interview studies conducted by Macey *et al*.; Ghanizadeh; Rafique and Hunt; and Díaz‐Atienza and Gurpegui provided in‐depth insights into the lived experiences, coping mechanisms and psychosocial challenges faced by individuals with AA. These studies highlighted themes including loss of identity, social stigma, maladaptive coping mechanisms and the significance of environmental stress. Overall, these studies collectively emphasise the profound psychosocial impact of paediatric AA, which includes impaired HRQL, emotional distress, social challenges and the need for comprehensive support and intervention strategies, in hopes of addressing the diverse range of psychosocial needs in these populations.

## Discussion

This systematic review identified 10 published studies on the psychosocial impact of AA in paediatric and adolescent populations. Four qualitative interview studies provided insight into the variability of patients who may experience the psychosocial impacts of AA and their unique presentations and manifestations.^10^, ^12^, ^13^, ^14^ The findings of the accepted studies collectively demonstrate the distinct psychosocial impacts of AA on paediatric and adolescent populations, which include impaired quality of life, increased levels of anxiety and elevated rates of depression. The studies highlight that the manifestations and impact on quality of life can vary depending on the severity of the condition and individual patient‐specific factors. The results of this systematic review align with prior studies conducted on the same topic, which have consistently shown the negative psychosocial consequences of AA in children and adolescents.

It is also crucial to recognise and compare the psychosocial impact of AA with other dermatological conditions in paediatric and adolescent populations. One study identified that comparing children with AA to those with other dermatological conditions, epilepsy and vitiligo generally reported no significant differences in anxiety, however, observed that children with AA had higher scores for worry, oversensitivity and concentration issues compared to patients with other conditions.^17^ Additionally, severity of AA and psychological impact is potentially correlated. One prospective study included 153 patients with AA, with a mean age of 11.0 years (range 1.4–22.3).^18^ The Severity of Alopecia Tool (SALT) scores averaged 38.2, indicating varying degrees of disease severity: 54% had mild, 24.9% had moderate and 28.1% had severe disease.^18^ Of these patients, 47 completed the CDLQI, with an average score of 4.4, showing notable impacts on physical symptoms and self‐consciousness.^18^ Statistical analysis revealed that SALT scores were positively correlated with Family Dermatology Life Quality Index (FDLQI) scores (*P* < 0.001) and negatively correlated with Quality of Life in Chronic Child's Disease Questionnaire (QLCCDQ) per‐item and emotional domain scores (*P* = 0.001).^18^ Duration of disease negatively correlated with QLCCDQ emotional domain scores (*P* = 0.049), while age of the child showed negative correlations with QLCCDQ scores but not with FDLQI scores.^18^

Through this systematic review, new data and insights have been gained regarding the psychosocial impact of AA. The studies have provided a comprehensive understanding of the challenges faced by paediatric and adolescent patients, including the negative effects on self‐esteem, the influence of familial and school‐related stress and the association with psychiatric comorbidities. Additionally, the qualitative interview studies have shed light on the emotional distress, maladaptive coping mechanisms and social stigma experienced by individuals with AA. Further research is necessary to better elucidate the relationship between psychosocial manifestations of AA in these populations, which can be accomplished by conducting large‐scale and longitudinal studies with diverse populations, providing a deeper understanding of the long‐term psychosocial outcomes and the efficacy of interventional approaches in addressing these outcomes.

Limitations of the current study include the use of a single reviewer for article screening and data extraction. The current study also included a limited number of heterogeneous records. Despite limited data, this review provides clinicians with an up‐to‐date compilation of all existing evidence that may prove useful when considering a holistic approach to paediatric and adolescent populations with AA. Moreover, increasing awareness of the link between AA and its psychosocial impact in these populations may ultimately increase the number of published reports in the literature.

## Conclusion

The psychosocial impact of AA in paediatric and adolescent populations is evident, contributing to negative effects on self‐esteem, body image, quality of life and social interactions. The need for comprehensive care addressing both the physical and emotional well‐being of these patients is crucial. Further research is required to focus on effective interventions and long‐term outcomes of psychosocial support. Collaborative efforts among health‐care professionals, psychologists, educators and support organisations are essential to develop holistic approaches for managing the psychosocial impact of AA in these age groups.
